# Advanced Bayesian BMD-Derived Genome-Wide Polygenic Scores Enhance Clinical FRAX-Based Fracture Risk Prediction in Postmenopausal Women

**DOI:** 10.1007/s00223-026-01497-8

**Published:** 2026-03-24

**Authors:** Anqi Liu, Jianing Liu, Qing Wu

**Affiliations:** Department of Biomedical Informatics, College of Medicine, The Ohio State University, 250 Lincoln Tower, 1800 Cannon Drive, Columbus, OH 43210 USA

**Keywords:** Fracture risk, Polygenic risk score, Bayesian analysis, Osteoporosis, Genome-wide association study, Post-menopause, Bone density, Genetic predisposition to disease, Risk assessment, Medical decision making

## Abstract

Current fracture prediction tools, including the Fracture Risk Assessment Tool (FRAX), rely primarily on clinical risk factors and bone mineral density measurements, yet omit genetic information. Given osteoporosis's substantial heritability (estimated at 50–80%), excluding genetic data may lead to clinical misclassification and uncertainty, particularly among postmenopausal women near clinical intervention thresholds. This study aimed to enhance clinical fracture risk prediction by integrating advanced Bayesian-derived genome-wide polygenic scores (GPS) into FRAX (Bayesian GPS-FRAX). Using genetic data from the Genetic Factors for Osteoporosis Consortium (GEFOS), we derived GPS using two advanced Bayesian methods, Polygenic Risk Score Continuous Shrinkage (PRS-CS) and Summary-data-based Bayesian Regression (SBayesR), selected for their ability to model complex genetic architectures and linkage disequilibrium. Bayesian GPS was integrated into FRAX, creating the Bayesian GPS-FRAX model, which was subsequently validated in 6932 postmenopausal women from the Women’s Health Initiative. We evaluated model performance in terms of both discrimination and clinical utility, with clinical utility primarily assessed using net reclassification improvement (NRI), threshold-based clinical reclassification, and decision curve analysis. Robustness and generalizability were confirmed through independent validation in a separate cohort of 3688 women. Bayesian GPS-FRAX modestly improved discrimination, increasing the time-dependent AUC from 0.72 to 0.74, and yielded clinically meaningful gains in risk classification, with a NRI of 4.55–5.07% compared with FRAX-CRF. Approximately one-third of reclassified individuals had baseline FRAX scores within 15–25%, a critical zone of clinical uncertainty. Bayesian GPS-FRAX successfully reclassified these women, correcting misclassification and enabling more accurate and timely clinical interventions. The greatest reclassification benefit was evident among women older than 70. Decision curve analyses demonstrated consistent net clinical benefit across clinically relevant thresholds (15–25%), notably at the widely used 20% treatment threshold. Independent validation further confirmed robust generalizability and clinical applicability. Integrating Bayesian GPS into FRAX substantially enhances discrimination and clinical fracture risk stratification, especially in older women and individuals near critical intervention thresholds. By addressing genomic complexity and clinical uncertainty through advanced Bayesian methodologies, Bayesian GPS-FRAX represents a meaningful advancement toward personalized osteoporosis management. Prospective validation in diverse cohorts, along with assessments of clinical implementation and cost-effectiveness, will be essential to facilitate the integration of this precision-based approach into routine clinical practice.

**Supplementary Information:**

The online version contains supplementary material available at 10.1007/s00223-026-01497-8.

## Introduction

Osteoporosis, characterized by reduced bone density and compromised skeletal microarchitecture, is a growing global health concern due to aging populations and the rising incidence of fragility fractures. In the United States alone, approximately 10 million adults aged 50 years or older have osteoporosis, with an additional 34 million at elevated risk, collectively resulting in nearly 1.5 million osteoporotic fractures annually [1]. These fractures significantly increase morbidity, mortality, healthcare expenditures, and profoundly impair patient quality of life [2]. Enhancing the accuracy of fracture prediction tools to facilitate precise clinical intervention remains an urgent public health priority.

Current clinical fracture prediction tools, notably the Fracture Risk Assessment Tool (FRAX), rely primarily on clinical risk factors and bone mineral density (BMD) but exclude genetic information. Given the substantial heritability of osteoporosis, estimated at approximately 50–80% [3–5]; the omission of genetic data significantly contributes to misclassification, clinical uncertainty, and suboptimal treatment decisions. This limitation is particularly pronounced among postmenopausal women who frequently fall into clinical uncertainty or "gray zones" near intervention thresholds [6–8].

Previous efforts to enhance fracture risk prediction using genome-wide polygenic scores (GPS) or polygenic risk scores (PRS) have yielded modest clinical benefits due to methodological constraints, such as inadequate modeling of polygenicity and linkage disequilibrium [9–13]. Most previous models have demonstrated only marginal improvements in predictive accuracy and limited impact on treatment decisions. Typically, these studies evaluated improvement through the area under the receiver operating characteristic curve (AUC) [11, 14]. However, as previously noted [15, 16], small improvements in AUC may underestimate the clinical value of novel predictors, especially when added to well-calibrated tools like FRAX. Because AUC summarizes global discrimination across the entire risk spectrum, it may be relatively insensitive to improvements occurring near clinically relevant decision thresholds. Therefore, in addition to AUC, we evaluated clinical utility using complementary metrics, including net reclassification improvement (NRI) and decision curve analysis (DCA), which more directly assess changes in risk classification and potential clinical benefit relevant to clinical decision-making [17, 18].

To overcome these methodological limitations, we developed a genome-informed fracture risk assessment tool (Bayesian GPS-FRAX) integrating advanced Bayesian genome-wide polygenic scoring methodologies into the established fracture risk assessment framework. Specifically, we employed Polygenic Risk Score Continuous Shrinkage (PRS-CS) and Summary-data-based Bayesian Regression (SBayesR), methods selected for their robust capability to address genetic complexity and linkage disequilibrium [19, 20]. Our primary objective was to assess whether integrating Bayesian-derived GPS into fracture risk assessment could improve individualized fracture risk stratification and clinical decision-making. We hypothesized that modeling genetic complexity using Bayesian methods would correct FRAX misclassifications near treatment thresholds and enable actionable improvements in clinical care.

## Materials and Methods

### Study Design and Population

This retrospective cohort study utilized clinical and genetic data from postmenopausal women enrolled in the Women’s Health Initiative (WHI), a large, multi-center study designed to investigate major causes of morbidity and mortality in postmenopausal women in the United States. Between 1993 and 1998, 161,808 postmenopausal women aged 50–79 years were recruited across 40 WHI clinical centers nationwide. Recruitment strategies included population-based mailings, community outreach, media campaigns, and direct engagement with clinical practices. Eligible participants were required to be postmenopausal, aged 50–79 years, expected to remain in the same geographic area for at least three years, and willing to provide written informed consent. Women were excluded if they had medical conditions likely to limit survival to less than three years, conditions impairing study participation or adherence, or other contraindications related to specific WHI clinical trial components.

**Fig. 1:**
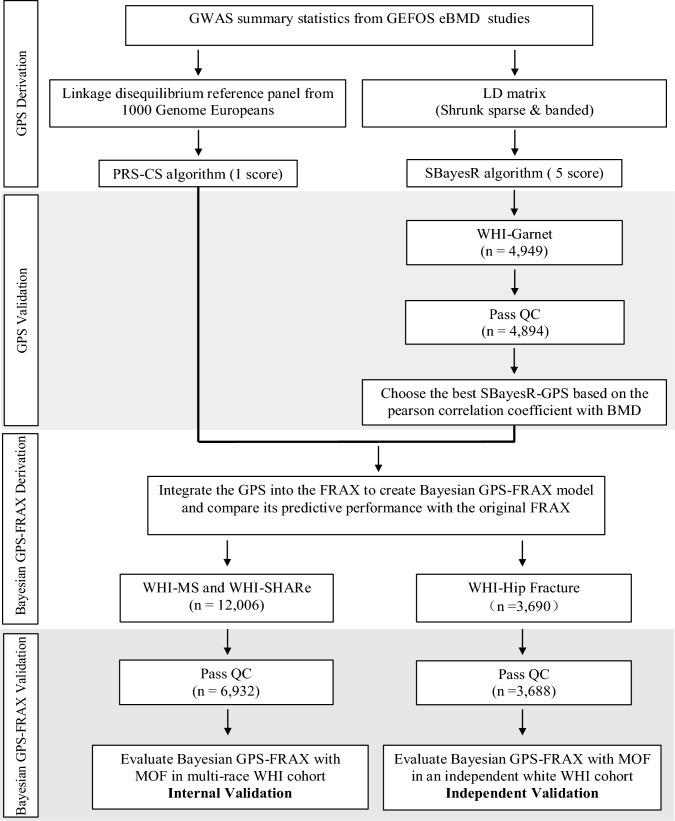
Workflow for Bayesian Genome-wide Polygenic Score (GPS) Derivation, Validation, and Integration into FRAX.GPS were derived from genome-wide variants from GEFOS eBMD data. PRS-CS (continuous shrinkage Bayesian priors) generated one GPS; SBayesR(Summary-data-based Bayesian Regression) generated five GPS. The optimal GPS was selected based on highest correlation with bone mineral density (BMD) in WHI-GARNET (n = 4894 after QC), integrated into FRAX (Bayesian GPS-FRAX), internally validated in WHI-MS and WHI-SHARe (n = 6932), and independently validated in a non-overlapping WHI Hip Fracture cohort (n = 3688). Key QC criteria: genotype call rate ≥ 95%, Hardy–Weinberg equilibrium *P* ≥ 1 × 10–6, minor allele frequency ≥ 0.01. Detailed Bayesian parameters in eMethod1

For the analysis, we included participants from the WHI Genomics and Randomized Trials Network (WHI-GARNET), WHI Memory Study (WHI-MS), WHI SNP Health Association Resource (WHI-SHARe) and WHI Hip Fracture GWAS (WHI Hip-Fracture). Data were accessed via the Database of Genotypes and Phenotypes (dbGaP; accession number phs000200.v12. p3). Participants were eligible if genetic data, complete clinical variables, and documented follow-up fracture outcomes were available. Explicit exclusion criteria included participants with > 20% missing clinical data or missing fracture outcome data. The final analytic sample comprised 6932 postmenopausal women (Fig. 1).

### Fracture Ascertainment and Clinical Data

Fracture outcomes encompassed Major Osteoporotic Fracture (MOF), defined as fractures of the hip, spine, wrist, or proximal humerus. Fractures were ascertained by participant self-report, validated previously within the WHI cohort [21]. Participants were followed for an average of 12 years (range, 1.0–22.5 years) from baseline, with follow-up calculated from enrollment or randomization until the first fracture event or death. FRAX provides a 10-year probability of major osteoporotic fracture; therefore, we aligned the observed outcome to a 10-year time horizon by defining the endpoint as incident MOF within 10 years of baseline, ensuring that calibration and clinical utility metrics are directly interpretable for 10-year decision support. Deaths occurring before 10 years were treated as censoring in time-to-event analyses. As FRAX accounts for competing mortality, differences in the handling of death may modestly influence absolute risk calibration, particularly among older women. Clinical risk factors included demographic and medical information collected at baseline. Age was recorded at enrollment; height and weight were measured using calibrated stadiometers and digital scales; prior osteoporosis history, parental hip fracture, and glucocorticoid use were assessed via standardized, self-administered WHI questionnaires; and rheumatoid arthritis was obtained from physician-diagnosed medical history forms. Hip BMD was measured in a subset of participants using dual-energy X-ray absorptiometry (DXA) with calibrated Hologic densitometers.

### Genotype Data and Quality Control

Genotyping was conducted using Illumina and Affymetrix platforms, with imputation using the Michigan Imputation Server with the 1000 Genomes Project (Phase 3 European reference panel) [22]. Rigorous quality control included sample call rates ≥ 95%, SNP call rates ≥ 90%, Hardy–Weinberg equilibrium (*p* ≥ 1 × 10^−6^), and minor allele frequency (MAF ≥ 0.01). Genotype QC was conducted using PLINK [23].

### Genome-Wide Polygenic Score (GPS) Calculation

To calculate GPS, we utilized publicly available summary statistics from the 2018 release of the GEnetic Factors for OSteoporosis (GEFOS) Consortium, based on genome-wide association analyses of estimated BMD (eBMD) from the UK Biobank [24]. eBMD is an ultrasound-derived estimate of bone density obtained using quantitative heel ultrasound, measured at the calcaneus (heel) via the Sahara Clinical Bone Sonometer (Hologic Inc). The phenotype is computed using speed of sound (SOS) and broadband ultrasound attenuation (BUA) parameters and is widely used in the UK Biobank osteoporosis GWAS. Importantly, eBMD shows a high genetic correlation with DXA-derived BMD, supporting its validity as a genetically informed proxy for skeletal fragility [25]. The discovery dataset comprised 13,753,401 SNPs from the GEFOS eBMD GWAS. GPS were calculated using two Bayesian methods: Polygenic Risk Score Continuous Shrinkage (PRS-CS) and Summary-data-based Bayesian regression (SBayesR). PRS-CS employs continuous shrinkage prior on SNP effect sizes, incorporating linkage disequilibrium (LD) structure from a 1000 Genomes European reference panel, robustly capturing fine-scale LD patterns and improving predictive accuracy [26].

1 $$ y = X\beta + \epsilon ,\epsilon \sim {\mathcal{N}}\left( {0,\sigma^{2} I} \right){\mathrm{,GPS}}_{{{\text{PRS - CS}},i}} = \mathop \sum \limits_{j = 1}^{M} \widehat{{\beta_{j} }}G_{ij} $$

2 $$ \widehat{{\beta_{j} }} \sim {\mathcal{N}}\left( {0,\phi \psi_{j} } \right),\psi_{j} \sim {\mathrm{Gamma}}\left( {a,b} \right) $$

PRS-CS employs a continuous shrinkage prior on SNP effect sizes, combining global ($\phi$) and local ($\psi_{j}$) shrinkage parameters to adaptively control sparsity and polygenicity. Posterior effects $\widehat{{\beta_{j} }}$ were inferred using a 1000 Genomes European LD reference panel and a fully Bayesian Gibbs sampler.

SBayesR models SNP effects with a mixture of normal distributions, directly integrating LD structures through LD matrices. This method effectively addresses polygenic complexity and localized LD correlation via optimized hyperparameters [12].

3 $$ y = X{\upbeta } + \epsilon ,\epsilon \sim {\mathcal{N}}\left( {0,\sigma_{e}^{2} I} \right), {\mathrm{GPS}}_{{{\mathrm{SBayesR}},i}} = \mathop \sum \limits_{j = 1}^{M} \widehat{{\beta_{j} }}G_{ij} $$

4 $$ {\upbeta }_{j} \sim \pi_{1} \delta_{0} + \pi_{2} {\mathcal{N}}\left( {0,\gamma_{2} \sigma_{\beta }^{2} } \right) + \pi_{3} {\mathcal{N}}\left( {0,\gamma_{3} \sigma_{\beta }^{2} } \right) + \pi_{4} {\mathcal{N}}\left( {0,\gamma_{4} \sigma_{\beta }^{2} } \right) $$

It models SNP effects as a mixture of four normal distributions with variance components $\gamma_{k} \sigma_{\beta }^{2}$ and mixture proportions $\pi_{k}$. The posterior SNP effects $\widehat{{\beta_{j} }}$ were estimated using summary-level LD matrices and Gibbs.

### Bayesian GPS-FRAX Model Development

Bayesian GPS-FRAX integrated GPS into FRAX by using the 10-year major osteoporotic fracture probability estimated by the FRAX tool based on clinical risk factors alone (CRF) as the baseline predictor. FRAX-CRF represents the FRAX-calculated 10-year fracture probability derived from clinical risk factors which includes age, sex, BMI, prior fracture, parental hip fracture, current smoking, alcohol use, glucocorticoid exposure, rheumatoid arthritis, and secondary osteoporosis, without inclusion of BMD or genetic information. To minimize confounding due to population stratification, we included the first ten principal components (PCs) of genetic ancestry, computed using EIGENSTRAT, as covariates in all GPS-adjusted models. Let ${\mathrm{P}}_{{{\mathrm{FRAX}}}}$ denote the 10-year FRAX MOF probability. The Bayesian GPS-FRAX model is:

5 $$ \begin{aligned} {\mathrm{log}}h({\mathrm{t}}|{\mathrm{X}}) & = \log h_{0} \left( t \right) + {{\upbeta }}_{1} {\mathrm{*log}}\left( {\frac{{P_{{FRAX}} }}{{1 - P_{{FRAX}} }}} \right) + {{\upbeta }}_{2} \cdot {\mathrm{GPS}} \\ & \quad + {{\upbeta }}_{3} \cdot {\mathrm{Age}} + {{\upbeta }}_{4} \cdot \left( {{\mathrm{GPS}} \cdot {\mathrm{Age}}} \right) + \sum \limits_{{k = 1}}^{{10}} r_{k} PC_{k} \\ \end{aligned} $$

Although age is included in the original FRAX calculation, we retained age as an independent covariate and incorporated the GPS × age interaction term to account for potential residual confounding and to capture the age-dependent genetic effect on fracture risk. This adjustment ensures that the genetic contribution is evaluated independently of the age weighting embedded within FRAX. Separate models were developed using PRS-CS and SBayesR-derived GPS.

### Statistical Analysis

Baseline characteristics were summarized using means (standard deviation, SD) or frequencies (percentages). Between-group comparisons were performed using chi-square, t-tests, or Fisher’s exact test as appropriate.

Calibration performance was evaluated using regression-based calibration intercept and slope derived from Cox proportional hazards models. The calibration slope was estimated by refitting a Cox model with the original linear predictor as the sole covariate, and the intercept was obtained by including the linear predictor as an offset term. Bootstrap resampling (1000 repetitions) was used to obtain optimism-corrected estimates and 95% confidence intervals. In each bootstrap iteration, the model was refitted in the resampled dataset and its calibration performance was evaluated both in the bootstrap sample and in the original cohort. The mean difference between these two performance estimates across 1000 iterations, which reflects overfitting, was subtracted from the apparent calibration to obtain bias-corrected estimates, following standard bootstrap validation procedures [27].

Discrimination performance including time-dependent AUC at year 10 and C-index was evaluated. Time-dependent AUC at year 10 was evaluated using stratified fivefold cross-validation. It was performed within the primary WHI analytic cohort (WHI-MS, WHI–SHARe), in which the dataset was randomly partitioned into five folds while preserving the proportion of fracture events in each fold. Four folds were used for model training and one-fold for testing, iterating across all five partitions to ensure non-overlapping training and validation sets.

Decision Curve Analysis (DCA) quantified the net clinical benefit of Bayesian GPS-FRAX models compared to FRAX-CRF, assessing clinical utility across fracture probability thresholds. Decision thresholds were pre-specified at 10%, 20%, and 30% 10-year MOF risk; 20% is the U.S. National Osteoporosis Foundation(NOF) [13] treatment threshold.

Treatment eligibility-based reclassification was performed using two guideline-based thresholds. A fixed 20% threshold was applied based on the NOF, and Age-dependent thresholds from the UK National Osteoporosis Guideline Group (NOGG) [28] were also evaluated.

### Sensitivity and Subgroup Analyses

The Subgroup analyses stratified participants by age (≤ 70 vs. > 70 years), based on established clinical fracture risk patterns and guideline-based age-dependent intervention thresholds that demonstrate a marked acceleration in fracture risk beyond age 70 [29, 30]. Sensitivity analyses compared Bayesian GPS-FRAX performance with and without hip BMD data to assess model robustness.

### Independent Validation

Independent validation was conducted in a cohort of 3688 White postmenopausal women (WHI–Hip Fracture, a sub-study of the Women’s Health Initiative), assembled separately from the development cohorts. Model performance was evaluated by assessing discrimination using time- dependent AUC and C-index. NRI was not evaluated in the independent validation cohort because participants exceeded this threshold, limiting the interpretability of category-based reclassification metrics.

### Software and Statistical Packages

All analyses were conducted using R software version 4.2.2 (R Foundation for Statistical Computing) [31]. We employed packages including "survival" version 3.8.3 (ROC analyses) [32], "nricens" version 1.6 (reclassification analyses) [33], "rmda" version 1.6 (decision curve analyses) [34] for this study.

### Ethical Considerations and Data Availability

All analyses were conducted under Institutional Review Board approval from Ohio State University (IRB approval number 2022H0420). Genotype and phenotype data are available via dbGaP under approved access agreements.

## Results

### Study Population Characteristics

The analytic cohort included 6932 postmenopausal women with a mean (SD) age of 64.2 (7.3) years; 409 (5.9%) experienced a MOF. Compared with participants without fractures, those with fractures were older (mean [SD] age: 69.1 [6.0] vs. 63.9 [7.3] years; *P* < 0.01), had lower body weight (mean [SD]: 72.8 [14.8] vs. 77.7 [16.4] kg; *P* < 0.01), and higher baseline FRAX-predicted 10-year MOF risk (mean [SD]: 13.5% [8.5] vs. 8.3% [6.9]; *P* < 0.01). Among participants with available hip BMD (n = 458), those with fractures had lower hip BMD compared with those without fractures (mean [SD]: 0.76 [0.12] vs. 0.89 [0.15] g/cm^2^; *P* < 0.01). GPS was derived from eBMD summary statistics, and by design is protective: higher values indicate greater BMD and correspond to lower fracture risk (hazard ratio per SD < 1) (Table 1).

**Table 1:** Baseline characteristics of 6932 women in the WHI independent testing cohort, stratified by major osteoporotic fracture (MOF) status

Characteristic	Participant cohort			
	Without MOF (n = 6523)	With MOF (n = 409)	Overall (n = 6932)	*P*-value^a^
Age (years), mean (SD)	63.89 (7.26)	69.13 (5.97)	64.20 (7.30)	< 0.01
Height (cm), mean (SD)	161.10 (6.17)	161.37 (6.57)	161.12 (6.19)	0.40
Race/Ethnicity				< 0.01
Black/African American	2356 (36.12%)	86 (21.03%)	2442 (35.23%)	
Hispanic/Latino	1038 (15.91%)	49 (11.98%)	1087 (15.68%)	
White	3129 (47.97%)	274 (67.00%)	3403 (49.09%)	
Weight (kg), mean (SD)	77.68 (16.41)	72.81 (14.77)	77.39 (16.36)	< 0.01
Hip BMD (g/cm^2^)^b^, mean (SD)	0.89 (0.15)	0.76 (0.12)	0.88 (0.15)	< 0.01
GPS (PRS-CS), mean (SD)	0.52 (0.14)	0.47 (0.14)	0.52 (0.13)	< 0.01
GPS (SBayesR), mean (SD)	0.50 (0.13)	0.47 (0.14)	0.49 (0.14)	< 0.01
Rheumatoid Arthritis, n (%)	234 (5.4%)	10 (6.4%)	244 (5.5%)	0.28
Parental hip fracture history, n (%)	1935 (29.66%)	162 (39.61%)	2097 (30.25%)	< 0.01
Glucocorticoid use, n (%)	6 (0.09%)	2 (0.49%)	8 (0.12%)	0.12
Previous osteoporosis, n (%)	355 (5.44%)	28 (6.85%)	383 (5.53%)	0.01
FRAX-predicted MOF risk (%), mean (SD)	8.31 (6.90)	13.54 (8.52)	8.62 (7.12)	< 0.01

*SD* standard deviation, *GPS(PRS)* Genome-Wide Polygenic Score derived by using Polygenic Risk Score Continuous Shrinkage, *GPS(SBayesR)* Genome-Wide Polygenic Score derived by using summary-data-based Bayesian Regression

^a^P-value was obtained by t-test for continuous variables, chi-square tests, and Fisher’s exact test (Glucocorticoid use) for the categorical variable

^b^Hip BMD available in a subset of participants (overall: n = 458, non-fracture: n = 420,fracture: n = 38)

### Model Robustness and Calibration

**Fig. 2:**
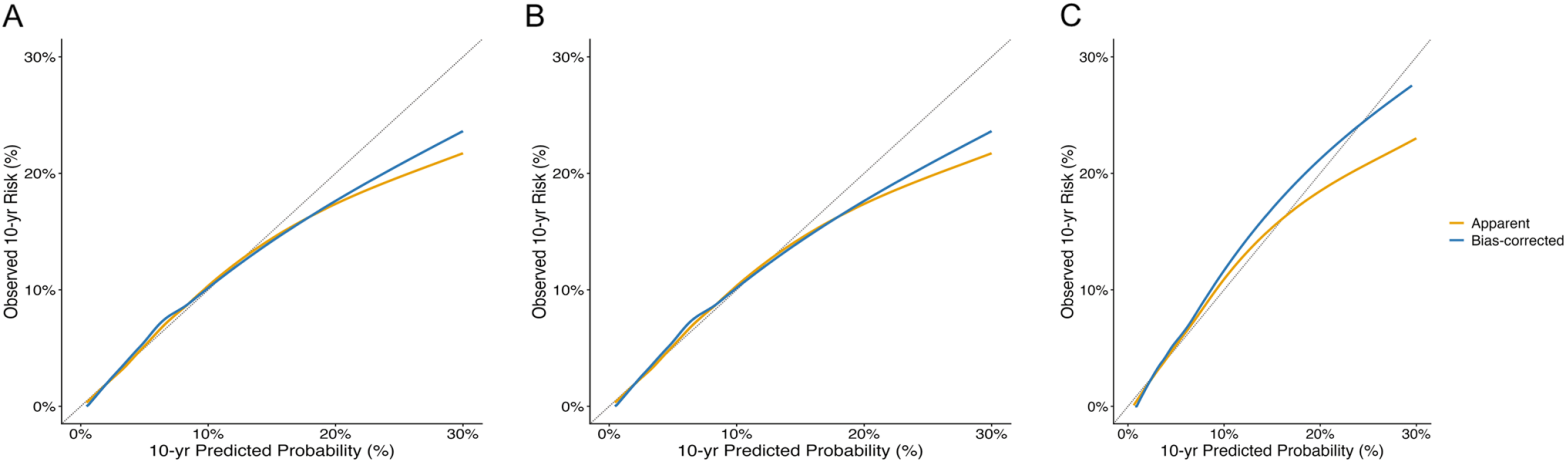
Calibration plots comparing Bayesian GPS-FRAX with FRAX-CRF. Calibration plots compare FRAX combined with PRS derived using SBayesR (**A**) and PRS-CS (**B**) with FRAX-CRF without genetic information (**C**). SBayesR and PRS-CS showed similar calibration patterns with minimal optimism after internal validation using 1000 bootstrap replications; however, standard FRAX-CRF demonstrated closer alignment with the ideal calibration line

Bayesian GPS-FRAX models incorporating PRS-CS and SBayesR demonstrated improved discrimination compared with FRAX-CRF, as reflected by higher time-dependent AUC and C-index values (eFigure 1 and eTable 1). Internal validation using fivefold cross-validation confirmed the robustness of these models, yielding consistent time-dependent AUC values across validation folds (PRS-CS: 0.754; SBayesR: 0.754) (eTable 2). Calibration analysis showed broadly comparable calibration between Bayesian GPS-FRAX models and FRAX-CRF after bootstrap correction, with minor differences observed across different regions of the predicted risk spectrum (Fig. 2).

### Clinical Reclassification and Net Reclassification Improvement (NRI)

Bayesian GPS-FRAX provided clinically relevant reclassification, achieving an NRI of approximately 4.55%-5.07% compared to FRAX-CRF. Approximately one-third (31%) of reclassified individuals had baseline FRAX scores between 15 and 25%, a clinically significant range near intervention thresholds. Overall, NRI was predominantly driven by improved classification of women who subsequently experienced fractures (fracture-event NRI: 5.38% for SBayesR, 5.87% for PRS-CS). The most pronounced clinical reclassification benefits occurred in women older than 70 years (NRI: 14.48% for SBayesR, 15.73% for PRS-CS), while less impact was observed in women ≤ 70 years (NRI: 2.06% for SBayesR, 1.67% for PRS-CS) (Table 2). In practical terms, Bayesian GPS-FRAX correctly identified approximately 24 additional fracture cases, compared to FRAX alone.

**Table 2:** Percentage reclassified and net reclassification improvement (NRI) for individual FRAX intervention criteria

	Fixed MOF 20%	Age-dependent MOF (NOGG guideline)
Reclassification (%)		
All subjects (SBayesR)	3.06%	2.97%
All subjects (PRS-CS)	3.03%	2.96%
Close to cut-off ^a^ (SBayesR)	31.22%	30.91%
Close to cut-off (PRS-CS)	31.22%	28.57%
Age ≤ 70 years (SBayesR)	2.43%	0.71%
Age ≤ 70 years (PRS-CS)	2.42%	0.73%
Age > 70 years (SBayesR)	9.17%	11.51%
Age > 70 years (PRS-CS)	9.17%	11.37%
Fracture outcome for NRI^b^ analysis(95% CI)		
NRI fracture, all subjects (SBayesR)	5.38% (2.90%, 8.12%)	4.89% (1.37%, 6.75%)
NRI fracture, all subjects (PRS-CS)	5.87% (3.20%, 8.97%)	5.38% (2.55%, 8.18%)
NRI non-fracture, all subjects (SBayesR)	− 0.83% (− 1.25%, − 0.41%)	− 0.87% (− 1.27%, − 0.47%)
NRI non-fracture, all subjects (PRS-CS)	− 0.80% (− 1.19%, − 0.43%)	− 0.83% (− 1.21%, − 0.45%)
NRI overall, all subjects (SBayesR)	4.55% (1.97%, 7.39%)	4.02% (1.37%, 6.75%)
NRI overall, all subjects (PRS-CS)	5.07% (2.31%, 8.11%)	4.55% (1.65%, 7.39%)
NRI overall, age < = 70 years (SBayesR)	2.06% (− 0.10%, 4.41%)	− 8.07% (− 14.62%, − 1.61%)
NRI overall, age < = 70 years (PRS-CS)	1.67% (0.56%, 4.13%)	− 7.46% (− 13.96%, 1.12%)
NRI overall, age > 70 years (SBayesR)	14.48% (7.66%, 22.21%)	6.42% (0.90%, 12.73%)
NRI overall, age > 70 years (PRS-CS)	15.73% (8.26%, 23.07%)	7.24% (0.92%, 13.75%)

^a^‘Close to cut-off’ indicates baseline FRAX within ± 5 percentage points of the threshold

^b^NRI computed at 10 years using nricens (time-to-event, censoring-adjusted) versus FRAX-CRF at the specified thresholds

### Diagnostic Accuracy and Clinical Threshold Reclassification

At the standard 20% treatment threshold, Bayesian GPS-FRAX (PRS-CS) reclassified 210 of 6932 women (3.0%), including 143 (2.1%) from low- to high-risk (fracture incidence: 21.0%) and 67 (1.0%) from high- to low-risk (fracture incidence: 9%). Similar results were observed with the SBayesR-based model (Table 3).

**Table 3:** Risk Reclassification between Standard FRAX and Bayesian GPS-FRAX and corresponding Fracture Incidence

Standard FRAX	Bayesian GPS-FRAX	N (PRS-CS)	Fracture (N, %)	N (SBayesR)	Fracture (N, %)
High	High	101	17 (16.8%)	100	17 (17%)
High	Low	67	6 (9.0%)	68	6 (8.8%)
Low	High	143	30 (21.0%)	144	28 (19.4%)
Low	Low	6621	356 (5.4%)	6620	358 (5.4%)

### Decision Curve Analysis (DCA)

**Fig. 3:**
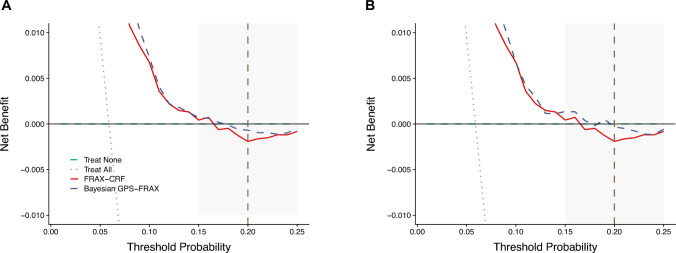
Decision curve analysis comparing the FRAX-CRF and Bayesian GPS-FRAX. The x-axis represents the threshold probability for starting treatment, and the y-axis represents the net benefit. Bayesian GPS-FRAX (dashed blue) consistently outperformed FRAX-CRF (solid red) between 15–25% thresholds, offering greater benefit for guiding treatment decisions. The “treat all” line (dotted grey) assumes all individuals are treated, while the “treat none” (dashed green) assumes no one is treated. The dashed vertical line marks the 20% clinical cutoff

Decision curve analysis demonstrated improved net clinical benefit for Bayesian GPS-FRAX compared to FRAX-CRF across clinically relevant risk thresholds (15%–25%), notably at the commonly utilized 20% intervention threshold. These findings illustrate the clinical utility and enhanced decision-making capacity provided by Bayesian GPS-FRAX (Fig. 3).

### Age-Stratified Analysis

Age-stratified analyses showed a clearer separation of observed fracture risk across GPS quantiles among women older than 70 years, indicating stronger genetic risk stratification in this age group (eFigure 2). Consistently, women older than 70 years demonstrated greater clinical utility, with improved time-dependent AUC (FRAX-CRF: 0.566; SBayesR: 0.658; PRS-CS: 0.656) and higher net clinical benefit across clinically relevant risk thresholds (15%–25%) (eTable 3, eFigure 3).

### Sensitivity Analysis Without BMD

Sensitivity analyses demonstrated robust predictive performance of Bayesian GPS-FRAX models without BMD (Time-dependent AUC: 0.80 with BMD vs. 0.76 without BMD for both models (eTable 4). Importantly, Bayesian GPS-FRAX models incorporating hip BMD achieved NRI values of 10.76% for the PRS-CS model and 11.72% for the SBayesR model relative to FRAX-CRF (eTable 4).

### Independent Validation

Independent validation in a cohort of 3688 White postmenopausal women demonstrated consistent results. Although absolute time-dependent AUC values were lower than those observed in the internal validation, reflecting cohort heterogeneity, Bayesian GPS-FRAX consistently maintained higher discrimination than FRAX-CRF (eTable 5).

## Discussion

In this cohort study of postmenopausal women, integrating Bayesian-derived GPS into FRAX (Bayesian GPS-FRAX) meaningfully improved fracture risk prediction beyond traditional clinical models. Bayesian GPS-FRAX achieved clinically relevant improvements in net reclassification improvement (SBayesR:4.55%; PRS-CS: 5.07%), particularly among women aged over 70 and those near critical intervention thresholds, who represent substantial clinical uncertainty.

Although prior studies incorporating GPS into fracture prediction models have generally reported modest AUROC improvements [11, 14], small changes in AUROC may underestimate the clinical value of novel predictors, particularly near critical clinical thresholds [15, 16, 35]. Therefore, we additionally evaluated decision curve and reclassification metrics to better characterize the clinical impact of Bayesian GPS-FRAX. Traditional GPS methods have struggled to adequately address complex genetic architectures, primarily due to limitations in modeling linkage disequilibrium and polygenicity [9–13]. In contrast, our Bayesian methods (PRS-CS and SBayesR) address these complexities by more effectively modeling polygenic effect-size distributions and linkage disequilibrium patterns, thereby providing more precise fracture risk predictions [19, 20].

Bayesian GPS-FRAX demonstrated measurable improvements in discrimination, with AUC increasing from 0.72 for FRAX-CRF to 0.74 for both PRS-CS– and SBayesR–based models. Clinically, Bayesian GPS-FRAX improved the classification of women who experienced fractures, with an event NRI of 5.87%, facilitating more accurate treatment decisions. This corresponds to approximately 24 additional fracture cases correctly identified that would have been missed by FRAX alone. For instance, a 66-year-old woman with a FRAX score just below the 20% treatment threshold could be accurately classified as high-risk based on her genetic profile, prompting timely intervention. Conversely, a patient with a marginally elevated FRAX score but low genetic risk might benefit from monitoring or lifestyle interventions rather than pharmacological treatment. These illustrative scenarios demonstrate the clinical value of genetically informed, individualized fracture risk assessments in routine patient care.

A notable strength of our study is its validation under two distinct clinical practice guidelines, the NOF (U.S.) and NOGG (U.K.), demonstrating consistent reclassification improvements. However, several limitations should be acknowledged. First, fracture outcomes relied on self-reported data, potentially introducing recall bias or misclassification despite prior validation. In addition, because FRAX estimates 10-year fracture probability in the presence of competing mortality, whereas deaths before 10 years were treated as censoring in our time-to-event analyses, differences in the handling of death may modestly influence absolute risk calibration, particularly among older women. Second, the cohort of independent validation predominantly included postmenopausal women of European ancestry, limiting generalizability to ethnically diverse populations. Third, reliance on existing GWAS summary statistics from European-ancestry populations and imputation using the 1000 Genomes European reference panel inherently constrains variant coverage and predictive precision. In addition, although Bayesian GPS-FRAX enhances fracture risk stratification, the incorporation of genome-wide polygenic information into routine clinical practice remains limited by feasibility constraints. Unlike FRAX-CRF, which relies only on readily available clinical variables, genetic testing requires additional cost, infrastructure, and data-processing capacity. Nevertheless, these barriers are gradually diminishing as genetic testing costs have decreased substantially in recent years and availability has expanded with broader insurance coverage [36, 37].

Future research should focus on the prospective validation of Bayesian GPS-FRAX in diverse populations and clinical settings. Additionally, economic evaluations and clinical implementation studies will be essential to support real-world translation and scalability [38]. Addressing these priorities will advance precision-based fracture risk assessment, enabling targeted and personalized prevention strategies for osteoporosis management.

## Supplementary Information

Below is the link to the electronic supplementary material.

Supplementary file1 (DOCX 940 KB)

## Data Availability

The individual-level data used in this study are available through controlled access from the database of Genotypes and Phenotypes (dbGaP) at https://www.ncbi.nlm.nih.gov/projects/gap/cgi-bin/study.cgi?study_id=phs000200.v12.p3. The genome-wide association summary statistics were obtained from the GEnetic Factors for Osteoporosis (GEFOS) Consortium and are publicly available at http://www.gefos.org/?q=content/data-release-2018.
