# Supplementary material for: Advanced Bayesian BMD-Derived Genome-Wide Polygenic Scores Enhance Clinical FRAX-Based Fracture Risk Prediction in Postmenopausal Women
Source: Calcif Tissue Int. 2026 Mar 24;117(1):46. doi: 10.1007/s00223-026-01497-8 (PMC13013207; doi:10.1007/s00223-026-01497-8)
Supplement: Supplementary file 1 — Supplementary file1 (DOCX 940 KB) [file 223_2026_1497_MOESM1_ESM.docx]

**Supplemental Content**

**eMethods 1. Prior Settings for Bayesian GPS Derivation Using SBayesR**

**eFigure 1.** Comparison of the predictive performance of FRAX-CRF and two Bayesian GPS-FRAX**.**

**eFigure 2. Association between Genetic Polygenic Score (GPS) quantiles and observed fracture rates.**

**eFigure 3. Decision curve analysis (DCA) stratified by age (≤70 and >70 years) comparing FRAX-CRF with Bayesian GPS-FRAX.**

**eTable 1. Model performance metrics**

**eTable 2.** Cross-validation of model discrimination

**eTable 3.** **Sensitivity and age-stratified subgroup analysis comparing discrimination (Time-dependent AUC) and net reclassification improvement (NRI) for FRAX-CRF and Bayesian GPS-FRAX models in women aged ≤70 years and >70 years**

**eTable 4**. Sensitivity Analysis of Predictive Performance of Bayesian GPS-FRAX Models with and without BMD

**eTable 5.**  Summarized Predictive Performance for Independent Validation

**eMethods 1. Prior Settings for Bayesian GPS Derivation Using SBayesR**

**SBayesR assumes a mixture of normal distributions for SNP effects, controlled by two key priors:**

- **π: the proportion of SNPs in each mixture component**
- **γ: the corresponding variance for each component**

**The following combinations were used:**

| **GPS ID** | **π Settings** | **γ Settings** |
| --- | --- | --- |
| **1** | **0.50, 0.25, 0.15, 0.10** | **0.0, 0.001, 0.01, 0.1** |
| **2** | **0.10, 0.30, 0.30, 0.30** | **0.0, 0.001, 0.01, 0.1** |
| **3** | **0.01, 0.19, 0.30, 0.50** | **0.0, 0.001, 0.01, 0.1** |
| **4** | **0.05, 0.15, 0.30, 0.50** | **0.0, 0.001, 0.01, 0.1** |
| **5** | **0.10, 0.20, 0.30, 0.40** | **0.0, 0.001, 0.01, 0.1** |

**Supplementary Figure**

**eFigure 1. Comparison of the predictive performance of FRAX-CRF and two Bayesian GPS-FRAX.** ROC curves in panels (A) and (B) compare FRAX-CRF with Bayesian GPS-FRAX constructed using PRS-CS and SBayesR methods, respectively.

**
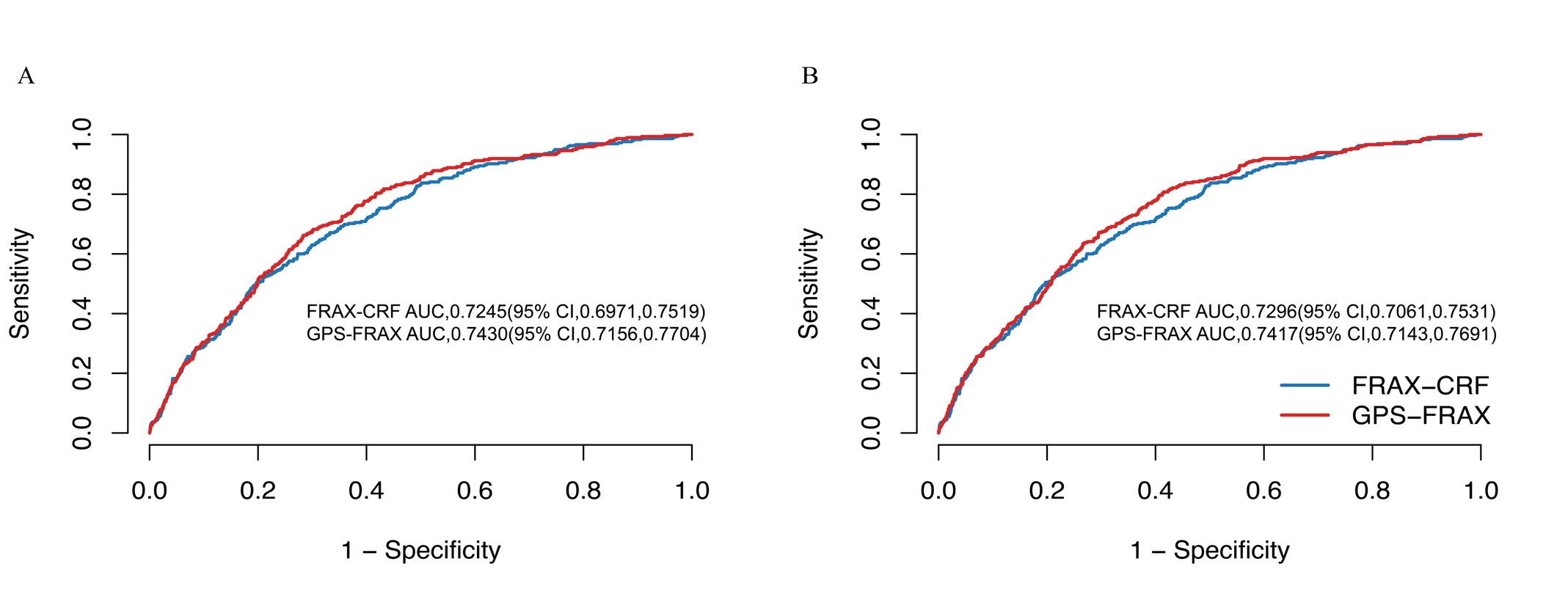
**

**eFigure 2. Association between Genetic Polygenic Score (GPS) quantiles and observed fracture rates. GPS quantiles categorize individuals based on where their genetic polygenic score lies within the population distribution. Lower GPS quantiles indicate higher genetic risk and are associated with a higher observed rate of major osteoporotic fractures. (A) Observed fracture rates stratified by GPS quantiles across age groups using PRS-CS-derived GPS. (B) Observed fracture rates stratified by GPS quantiles across age groups using SBayesR-derived GPS.**


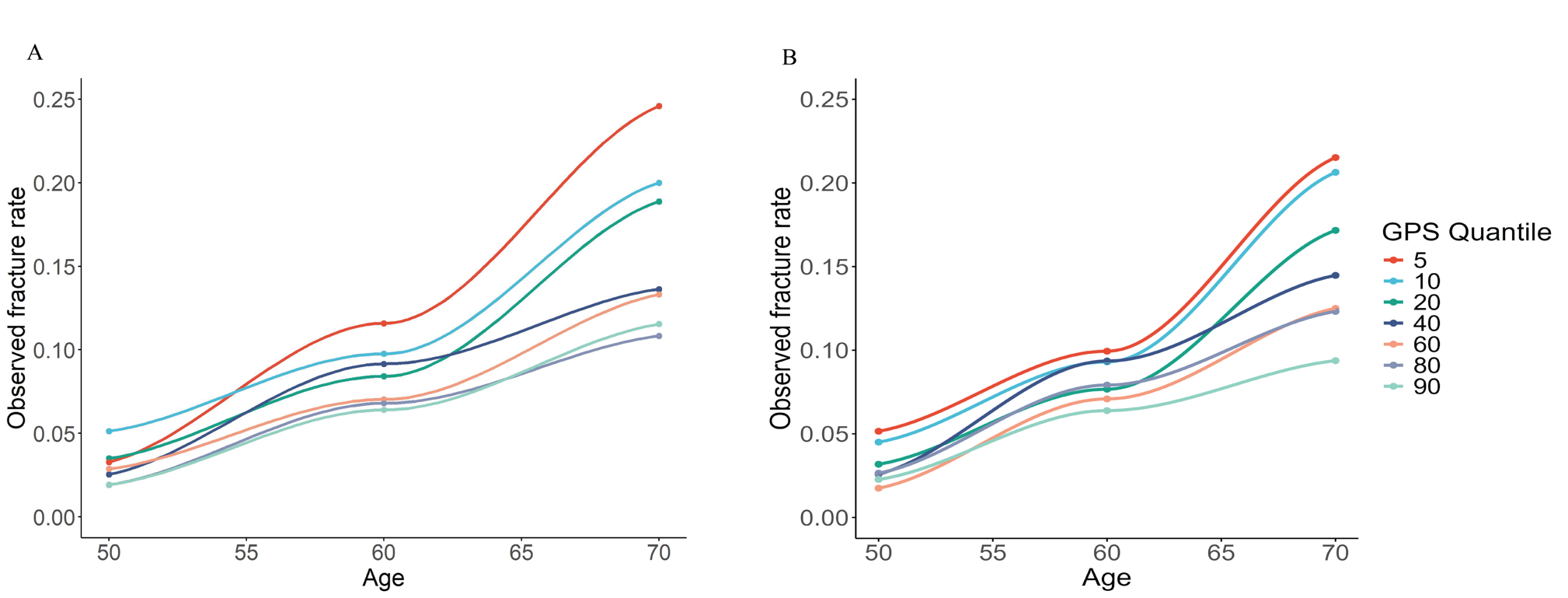


**eFigure 3. Decision curve analysis (DCA) stratified by age (≤70 and >70 years) comparing FRAX-CRF with Bayesian GPS-FRAX** **models.(A) Comparison between Bayesian GPS-FRAX(PRS-CS) and FRAX-CRF for participants aged ≤70 years.(B) Comparison between Bayesian GPS-FRAX(SBayesR) and FRAX-CRF for participants aged ≤70 years.(C) Comparison between Bayesian GPS-FRAX(PRS-CS) and FRAX-CRF for participants aged >70 years.(D) Comparison between Bayesian GPS-FRAX(SBayesR) and FRAX-CRF for participants aged >70 years. Dashed blue lines represent Bayesian GPS-FRAX models, solid red lines represent the FRAX-CRF model. The vertical dashed line marks the 20% intervention threshold.**


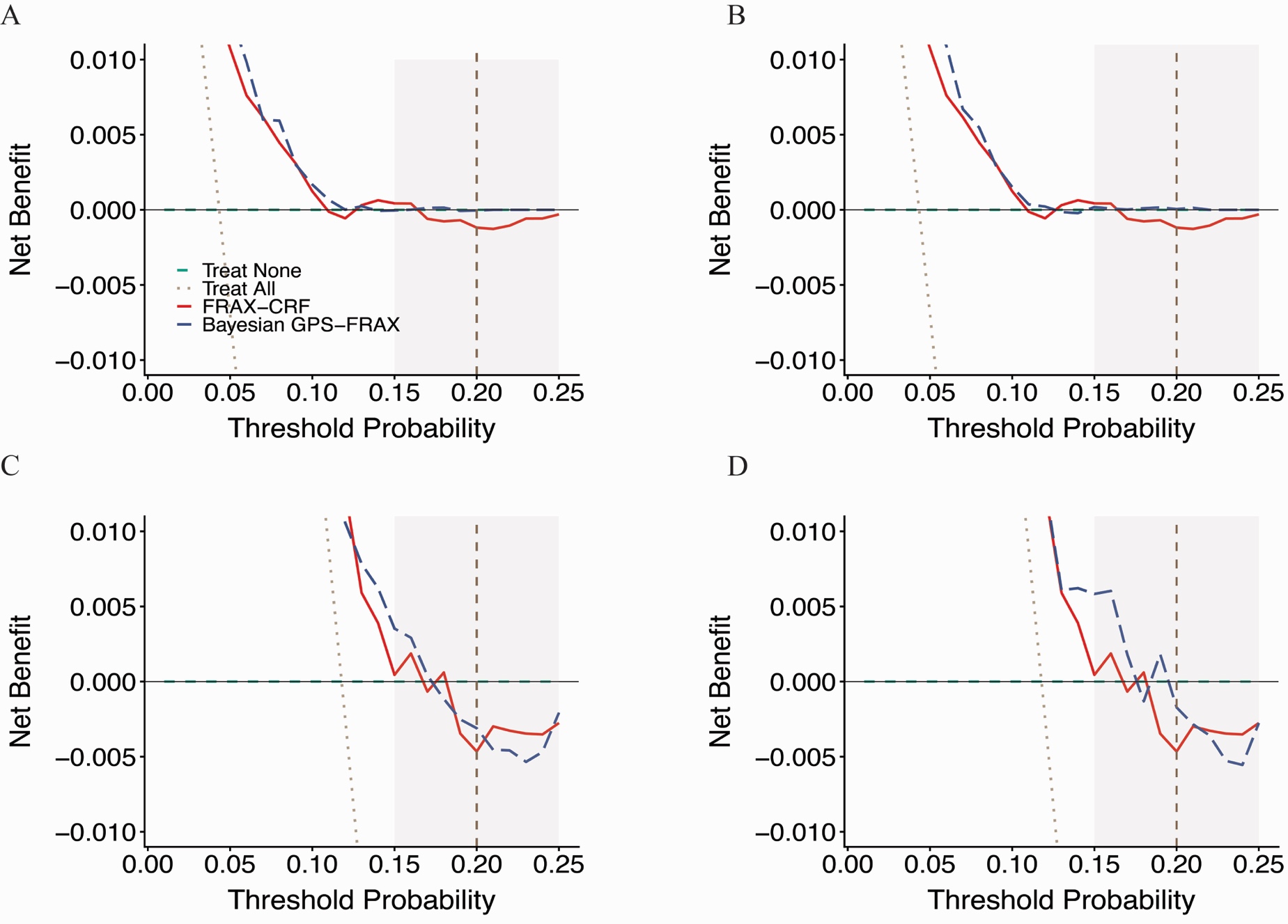


**Supplementary Table**

**eTable 1. Model performance metrics.**Summary of model discrimination, calibration, and reclassification performance for FRAX-CRF and Bayesian GPS-FRAX models. The Bayesian GPS-FRAX models incorporate genome-wide polygenic scores derived using PRS-CS and SBayesR methods. Metrics include time-dependent AUC (discrimination), C-index, NRI (reclassification), and calibration slope with 95% confidence intervals.

| Metric | FRAX-CRF | Bayesian GPS-FRAX(PRS-CS) | Bayesian GPS-FRAX(SBayesR) |
| --- | --- | --- | --- |
| AUC | 0.7245(0.6971,0.7519) | 0.7430(0.7156,0.7704) | 0.7417(0.7143,0.7691) |
| C-Index | 0.7118(0.6883,0.7350) | 0.7334(0.7116,0.7560) | 0.7325(0.7101,0.7548) |
| NRI | Reference | 5.07% | 4.55% |
| Calibration Slope | 1.0039(0.886,1.1304) | 0.9514(0.8534,1.0681) | 0.9515(0.8545,1.0639) |
|  |  |  |  |

**eTable 2.** **Cross-validation of model discrimination.** Time- dependent area under the receiver operating characteristic curve (AUC) values for the FRAX-CRF model and Bayesian GPS-FRAX models using polygenic scores derived via SBayesR and PRS-CS. Results are shown across five folds and as an overall mean from 5-fold cross-validation.

| Fold | AUC(FRAX-CRF) | AUC(PRS-CS) | AUC(SBayesR) |
| --- | --- | --- | --- |
| 1 | 0.786 | 0.794 | 0.795 |
| 2 | 0.731 | 0.759 | 0.760 |
| 3 | 0.708 | 0.731 | 0.728 |
| 4 | 0.699 | 0.717 | 0.716 |
| 5 | 0.732 | 0.771 | 0.769 |
| Mean | 0.732 | 0.754 | 0.754 |

**eTable 3.** **Sensitivity and age-stratified subgroup analysis** **comparing discrimination (Time-dependent AUC) and net reclassification improvement (NRI) for FRAX-CRF and Bayesian GPS-FRAX models in women aged ≤70 years and >70 years. Results are presented separately for fixed intervention threshold (MOF risk ≥20%) and age-dependent thresholds (based on NOGG guidelines).**

|  | Age ≤70 | | | Age>70 | | |
| --- | --- | --- | --- | --- | --- | --- |
|  | AUC | NRI  (Fixed MOF 20%) | NRI  (Age-dependent MOF) | AUC | NRI  (Fixed MOF 20%) | NRI  (Age-dependent MOF) |
| FRAX-CRF | 0.716 | Reference | Reference | 0.566 | Reference | Reference |
| Bayesian GPS-FRAX(PRS-CS) | 0.743 | 1.67% (0.56%,4.13%) | -7.46% (-13.96%,1.12%) | 0.656 | 15.73% (8.26%,23.07%) | 7.24% (0.92%,13.75%) |
| Bayesian GPS-FRAX(SBayesR) | 0.745 | 2.06% (-0.10%,4.41%) | -8.07% (-14.62%, -1.61%) | 0.658 | 14.48% (7.66%,22.21%) | 6.42% (0.90%,12.73%) |

Abbreviations: MOF, Major Osteoporotic Fracture ; NOGG, National Osteoporosis Guideline Group

**eTable 4**. **Sensitivity Analysis of Predictive Performance of Bayesian GPS-FRAX Models with and without BMD**

| Performance Metric | Bayesian  GPS-FRAX(PRS-CS)  (With BMD) | Bayesian  GPS-FRAX(PRS-CS) (Without BMD) | Bayesian  GPS-FRAX(SBayesR) (With BMD) | Bayesian GPS-FRAX(SBayesR) (Without BMD) |
| --- | --- | --- | --- | --- |
| AUC | 0.802 | 0.7646 | 0.804 | 0.7634 |
| NRI(%) | 10.76 |  | 11.72 |  |
| NRI (cases) | 10.52 |  | 10.52 |  |
| NRI (controls) | 0.24 |  | 1.19 |  |
|  |  |  |  |  |

Note: “With BMD” and “Without BMD” indicate whether hip BMD was included as a covariate in the Bayesian GPS-FRAX model.

All NRI estimates represent reclassification improvement of each Bayesian GPS-FRAX model relative to FRAX-CRF.

Analyses were conducted in 458 participants with available hip BMD.

**eTable 5. Summarized Predictive Performance for Independent Validation**

|  | FRAX-CRF | GPS-FRAX(PRS-CS) | GPS-FRAX(SBayesR) |
| --- | --- | --- | --- |
| AUC | 0.647(0.629,0.665) | 0.677(0.661,0.693) | 0.681(0.665,0.697) |
| C-index | 0.592(0.581,0.603) | 0.619(0.609,0.629) | 0.622(0.612,0.632) |
